# Determination of the Pharmacokinetics and Oral Bioavailability of Salicylamine, a Potent γ-Ketoaldehyde Scavenger, by LC/MS/MS

**DOI:** 10.3390/pharmaceutics2010018

**Published:** 2010-02-01

**Authors:** Irene Zagol-Ikapitte, Elena Matafonova, Venkataraman Amarnath, Christopher L. Bodine, Olivier Boutaud, Rommel G. Tirona, John A. Oates, L. Jackson Roberts, Sean S. Davies

**Affiliations:** 1 Division of Clinical Pharmacology, Departments of Pharmacology, Medicine, and Pathology, Vanderbilt University, Nashville, TN, USA; E-Mails: irene.zagol@vanderbilt.edu (I.A.Z.-I.); elena.matafonova@vanderbilt.edu (E.M.); venkataraman.amarnath@vanderbilt.edu (V.A.); christopher.l.bodine@vanderbilt.edu (C.L.B.); olivier.boutaud@vanderbilt.edu (O.B.); john.oates@vanderbilt.edu (J.A.O.); jack.roberts@vanderbilt.edu (L.J.R.); 2 Department of Physiology and Pharmacology, Schulich School of Medicine & Dentistry, University of Western Ontario, London, Ontario, Canada; E-Mail: Rommel.Tirona@schulich.uwo.ca (R.G.T.)

**Keywords:** levuglandins, isoketals, aldehydes, scavengers, oxidative stress, inflammation, lipid peroxidation, bioavailability, pharmacokinetics

## Abstract

Levels of reactive γ-ketoaldehydes derived from arachidonate increase in diseases associated with inflammation and oxidative injury. To assess the biological importance of these γ-ketoaldehydes, we previously identified salicylamine as an effective γ-ketoaldehyde scavenger *in vitro* and in cells*.* To determine if salicylamine could be administered *in vivo*, we developed an LC/MS/MS assay to measure salicylamine in plasma and tissues. In mice, half-life (t_1/2_) was 62 minutes. Drinking water supplementation (1-10 g/L) generated tissue concentrations (10-500 μM) within the range previously shown to inhibit γ-ketoaldehydes in cells. Therefore, oral administration of salicylamine can be used to assess the contribution of γ-ketoaldehydes in animal models of disease.

## 1. Introduction

Both inflammation and oxidant stress give rise to bicyclic endoperoxides (prostaglandin H_2_ and H_2_-isoprostanes, respectively) that non-enzymatically rearrange to form highly reactive γ-ketoaldehydes (γKA) [1,2]. These γKAs, termed levuglandins and isoketals, react extremely rapidly to covalently modify cellular proteins [2] and phosphatidylethanolamine [3]. Levels of γKA protein adducts increase in a number of conditions associated with inflammation and oxidative stress including Alzheimer’s Disease [4], atherosclerosis [5], myocardial infarction [6], end stage renal disease [5], sepsis [7], and hyperoxia [8]. Experiments with exogenously added γKAs in cell culture experiments suggest that formation of γKA protein adduct may contribute to disease pathogenesis [6,9,10,11,12,13,14,15]. To directly test whether formation of γKA protein adducts contribute to disease processes, development of selective inhibitors that block endogenous formation of γKA protein adducts *in vivo* is required. 

**Figure 1:**
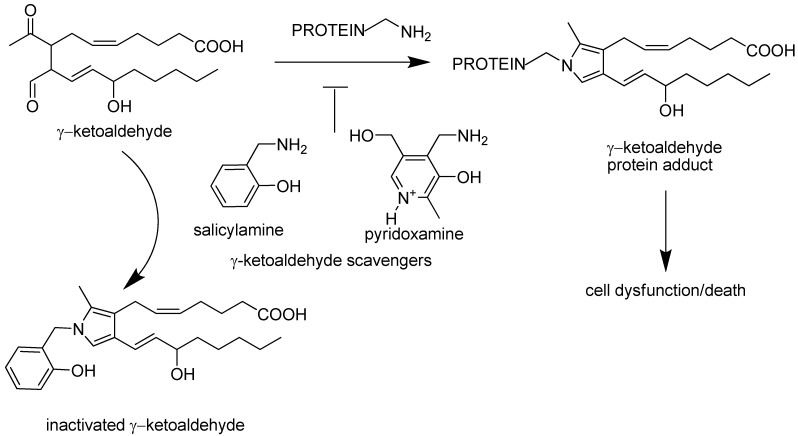
Arachidonate oxidation generates γ-ketoaldehydes that rapidly react with cellular proteins and can cause cell dysfunction and death. Scavengers such as salicylamine and pyridoxamine selectively react with γ-ketoaldehydes and thereby inhibit formation of γ-ketoaldehyde protein adducts, protecting against γ-ketoaldehyde induced cell dysfunction and cell death.

To this end, we recently characterized a class of phenolic amines including pyridoxamine (PM) and its lipophilic analogs such as salicylamine (SA) that inhibit protein modification by acting as γKA scavengers (Figure 1) and that preferentially react with γKAs over other lipid carbonyls produced by peroxidation such as 4-hydroxynonenal [15,16]. *In vitro*, the reaction rate of PM and its analogs with γKAs is more than a thousand times faster than the reaction rate of lysyl residues. However, as lipid peroxidation forms γKAs esterified in situ to phospholipids [13], lipophilic analogs are anticipated to be more efficacious than the highly hydrophilic PM. In keeping with this notion, the lipophilic PM analog SA was a better significantly better inhibitor of γKA protein adducts in cells than PM [15]. Additionally, SA protected HepG2 cells from cytotoxicity induced by hydrogen peroxide, while PM was ineffective [15]. Similarly, SA was able to protect against γKA and oxidant-induced sodium channel function inhibited by γKA or oxidants [17]. The protective effect of SA in cultured cells suggests that it may be a useful tool to study the biological importance of γKAs *in vivo* and to determine whether inhibiting γKAs is a useful therapeutic strategy for the treatment of disease. To facilitate *in vivo* studies, we developed an LC/MS/MS assay to measure SA levels in plasma and tissue and used this assay to explore the pharmacokinetics and oral bioavailability of SA in mice.

## 2. Experimental Section

### Synthesis of isotope-labeled salicylamine

All chemicals and solvents were purchased from VWR International (West Chester, PA) unless otherwise noted. [^2^H_6_]-Phenol (5 g, 50 mmol; Sigma-Aldrich, St. Louis, MO) and anhydrous MgCl_2_ (7.5 g), were dissolved in acetonitrile (150 ml), and triethylamine (26 ml) and paraformaldehyde (10.5 g) added. The suspension was refluxed for 1.5 h, cooled, and mixed with 4 M HCl (55 ml) on ice. It was extracted with ethyl acetate (4 × 25 ml) and the combined extracts were dried and evaporated. The crude product was purified by chromatography (silica gel, Fisher Scientific, Pittsburgh, PA; 3:1 hexane-ethyl acetate) and treated with hydroxylamine HCl (3.6 g) and sodium acetate (4.2 g). The resulting oxime was reduced in two batches (3.7 g) with zinc (6.5 g) and acetic acid (40 mL) at 10-25 °C for 3 h. The reaction mixtures were combined and filtered through a bed of Celite (Fisher). The filtrate was evaporated, then co-evaporated with toluene (20 ml) and ethanol (20 ml). [^2^H_4_]SA as acetic acid salt was crystallized from hot ethanol; yield 6.3 g (65%). [^14^C]phenol (250 μCi in 5 mmol; ViTrax Radiochemicals, Placentia, CA) was converted to [^14^C]SA in a similar manner. Formation of the expected product was confirmed by melting point analysis (186-187 ^o^C) and UV absorbance spectrum in comparison to previously reported values for salicylamine [18], as well as by mass spectrometry ([M+H]^+^ ion *m/z* 128) for [^2^H_4_]SA. Final purity was 98%. Unlabeled SA was prepared by reduction of salicylaldoxime (Fisher) with zinc and acetic acid. 

### Measurement of salicylamine lipophilicity

To measure the efficiency of extraction of SA by ethyl acetate, 5,000 dpm [^14^C]SA was added to 1 ml solutions at pH 5, 7, 7.4, 8, 8.5, 9, and 10. Ethyl acetate (4 ml) was then added, the two phases separated, and the amount of radiolabel in each determined by liquid scintillation counting. For measurement of PM extraction efficiency, 1 mM PM solution was prepared in PBS and absorbance measured at 325 nm. One ml aliquots of these solutions were then brought to pH 5, 7.4, and 9, and ethyl acetate (4 ml) added. The two phases were extracted, the ethyl acetate layer dried, resuspended in 1 ml PBS, and the absorbance of the aqueous layer and the ethyl acetate layer determined. 

### Animals

Twenty five C57BL6 male mice purchased from Jackson Labs (Bar Harbor, ME) and weighing 23-25 g were used for experiments. C57BL6 mice were chosen for these studies because they are the background strain for many transgenic mouse models of disease related to oxidative stress and inflammation. For pharmacokinetics studies, SA was dissolved in PBS at 36.8 g/L (200 mM) and 200 mg/kg injected intraperitoneally into ten C57BL6 mice. Two mice each were euthanized at 15, 30, 60, 120, and 240 minutes. Blood was collected, centrifuged, and the plasma layer removed and stored at -80 ^o^C until analysis. Liver, brain, kidney, heart were also collected and flash frozen in liquid nitrogen and then stored at -80 ^o^C until analysis. For oral bioavailability studies, solutions of SA were prepared from SA acetate salt at 1 g/L, 3 g/L, and 10 g/L in water. Solutions were transferred to red colored watering bottles to protect SA from photooxidation. Each of the C57BL6 mice were caged individually and given food and medicated water ad libitum. At the end of the feeding period, animals were euthanized in the morning and tissue and plasma collected. All procedures involved in the study were approved by the Vanderbilt’s Institutional Animal Care and Use Committee.

### Salicylamine assay

**Figure 2:**
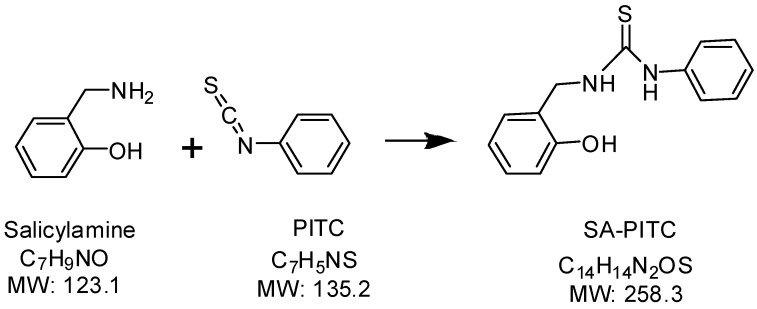
Reaction of PITC with salicylamine to form SA-PITC.

For plasma samples, 50 μl of the sample were added to 450 μl PBS containing 65 pmol of [^2^H_4_]SA. For tissue, the flash frozen organ was weighed, placed in 5 μl PBS, homogenized, and 65 pmoles of [^2^H_4_]SA was added to 500 μl of the homogenate for analysis. Phenyl isothiocyanate (PITC) working solution (2:2:6 PITC/triethylamine/acetonitrile v/v/v) was prepared fresh each day, and 30 μl of this solution, along with 500 μl acetonitrile, added to the sample to form SA-PITC (Figure 2). Samples were incubated at 60 ^o^C for 40 minutes in darkness. 

The derivatized samples were then extracted twice with 500 μl ethyl acetate, samples dried under nitrogen, and dissolved in 50 μl 20% methanol. Samples were analyzed by HPLC (ThermoFinnigan Surveyor MS pump; San Jose, CA) coupled to a triple quad mass spectrometer (ThermoFinnigan TSQ Quantum) using reversed phase chromatography (Magic Bullet C18 column 3A, Michrom BioResources, Auburn, CA) with the gradient programmed from 90% solvent A (water with 0.1% acetic acid) to 100% Solvent B (methanol with 0.1% acetic acid) over 4.0 minutes and then continuing at 100% B for an additional 1.0 minutes. Flow rate was 200 μl/min. Eluant was coupled directly to the mass spectrometer operated in multiple reaction monitoring (MRM) positive ion mode. Nitrogen was used for both the sheath and the auxiliary gases. The sheath and auxiliary gases were set to 49 and 15 (arbitrary units), respectively. The electrospray needle was maintained at 4000 V. The ion-transfer tube was operated at 35V and 250 °C. The tube lens voltage was set to 96 V. For analysis of underivatized SA and [^2^H_4_]SA, MRM was performed at *m/z* 124.1 ➔107.0 @-25 eV and *m/z* 128 ➔111.0 @-25 eV, respectively. These transitions result from loss of the amine from the parent compound. For SA-PITC and [^2^H_4_]SA-PITC, MRM were performed at *m/z* 259.1 ➔153.0 @-15 eV and m/z 263.1➔153.0 @-15 eV. These transitions result from fragmentation of the thioamide bond and the product ion represents the fragment derived from the PITC moiety. Additional MRM at *m/z* 259.1 ➔107.0 @-25 eV and *m/z* 263.1➔111.1@-25 eV for SA-PITC and [^2^H_4_]SA-PITC, respectively, for each product were also monitored for verification. These transitions result from fragmentation at the same thioamide bond as above, but the product ion represents the fragment derived from the SA moiety. The scan width for product ions was 0.5 μm and the cycle time for each ion was 0.05 s. The electron multiplier gain was set to 2 x 10^6^. The concentration of SA in plasma and tissue were calculated from the ratio of SA-PITC and [^2^H_4_]SA-PITC peak height.

### Pharmacokinetic Analysis

For data obtained from intraperitoneal SA administration, half-life (t_1/2_) was estimated by linear regression of the log-concentration *vs*. time profile of mean plasma levels between 30 and 240 min. The resulting slope furnished the elimination rate constant (k_e_) and t_1/2_ was calculated by:

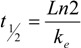
(1)


Apparent clearance (CL/F_app_) was calculated by obtaining the area under the SA plasma concentration – time curve (AUC) using the assumption that SA injected i.p. was fully bioavailable, (*i.e*., that the apparent fraction of absorbance (F_app_ ) was 1), with the following relationship:

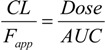
(2)


Apparent volume of distribution (V/F_app_), again assuming F_app_ was 1, was calculated by model-independent methods using the relationship:

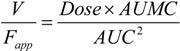
(3)
where AUMC is the area under the first moment (concentration × time) *vs*. time curve.

Calculations were performed in Microsoft Excel. All graphs were generated in GraphPad Prism version 4.03 software (La Jolla, CA). 

## 3. Results and Discussion

### Development of method to assay SA in biological tissue

**Figure 3:**
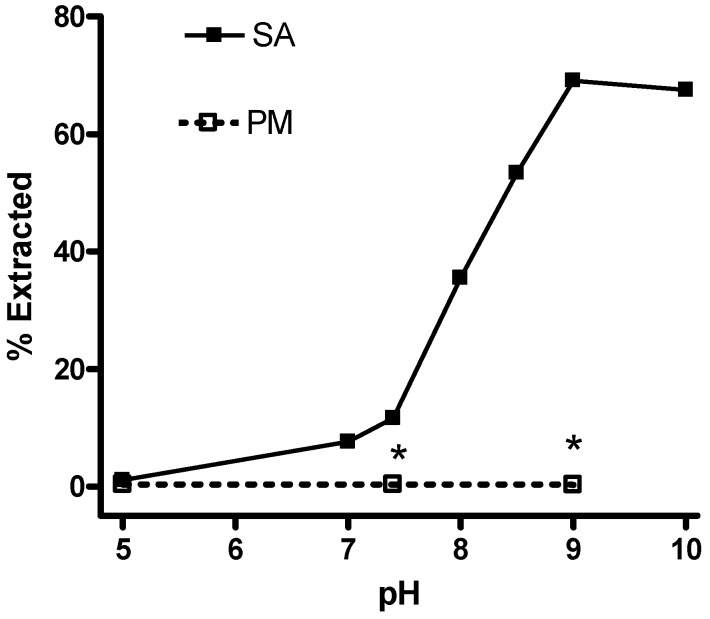
Salicylamine (SA) is more lipophilic than pyridoxamine (PM) and readily extracts into ethylacetate. [^14^C]SA was added to solution buffered from pH 5 to 10, four volumes of ethyl acetate added, and the amount of radiolabeled recovered in the aqueous and ethyl acetate layer determined by liquid scintillation counting. Similar experiments were carried out with unlabeled PM, using UV absorbance to detect PM levels. (* p < 0.001 *vs*. SA, t-test)

The greater bioactivity of SA compared to PM as a γKA scavenger has been ascribed to its potentially greater lipophilicity. The methyl amine moieties of both SA and PM have a pKa of 7.9, so that a portion of the methyl amine is uncharged (deprotonated) for both molecules at physiological pH. However, for pyridoxamine, the pyridine nitrogen remains positively charged (protonated) at physiological pH, so that its partitioning into lipid bilayers would be disfavored. To determine optimal extraction conditions for measurement of SA and to also directly compare lipophilicity of SA and PM, we measured the amount of SA and PM extracted into ethyl acetate at various pH. [^14^C]SA was spiked into solutions buffered at a range of pH, extracted with ethyl acetate, and percentage of [^14^C]SA in the two phases determined. At physiologically relevant pH 7.4, 15% of the SA partitioned into ethyl acetate, while less than 1% PM did so (Figure 3). Even at pH 9, less than 1% PM partitioned into ethyl acetate, while 69% of SA was found in the ethyl acetate layer. These results confirm the greater lipophilicity of SA compared to PM.

### LC/MS/MS measurement of SA in plasma and tissue.

**Figure 4:**
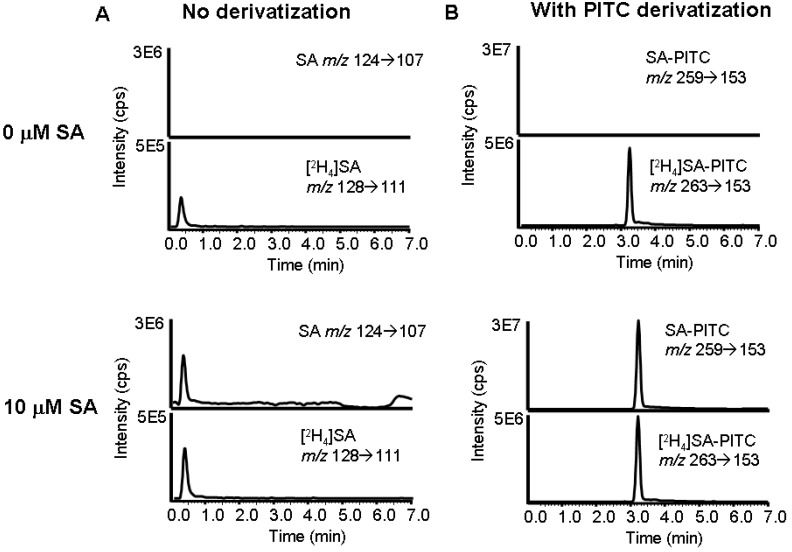
Analysis of underivatized SA or PITC derivatized SA by LC/MS/MS. **A)** 0 or 10 uM SA was spiked into 50 μl plasma, along with 65 pmol [^2^H_4_]SA, and then analyzed without derivatization by LC/MS/MS as described in the Experimental section. **B)** 0 or 10 uM SA was spiked into 50 μl plasma, along with 65 pmol [^2^H_4_]SA, and then subjected to PITC derivatization prior to analysis by LC/MS/MS as described in the Experimental section.

We have previously quantified SA during *in vitro* reactions by LC/MS/MS without any purification or derivatization [15]. We therefore determined if we could use this same LC/MS/MS method to measure SA in plasma using only ethyl acetate extraction at pH 9 for purification. Extraction was performed on 50 μl aliquots of plasma spiked with 0 or 10 μM SA (final concentration), along with 65 pmol [^2^H_4_]SA internal standard. The resulting chromatograms demonstrated that SA could be measured by this method, although the sensitivity was low (Figure 4A) (S/N = 9; peak height 1.6E6). We then assessed the effect of derivatization of SA with phenyl isothiocynanate (PITC) on sensitivity and chromatography. We found that overall sensitivity for PITC derivatized SA was greater (S/N = 498, peak height 2.9E7) compared to non-derivatized sample and the derivatized compound showed greater retention on the C18 column under our gradient conditions (Figure 4B). We therefore used PITC derivatization for all subsequent analysis. 

### Validation of LC/MS/MS method to measure SA

To determine the linearity of the assay, we prepared 10 nM to 1 mM solutions of SA in PBS and also in plasma. 50 ul aliquot of each sample were added to 65 pmoles [^2^H_4_]SA and the samples analyzed after PITC derivatization and extraction. Increasing concentrations of SA gave a linear response over all concentrations in PBS (*y* = 0.995 +0.008 R^2^ = 0.995). For plasma, the lower limit of detection (S/N > 3) was approximately 100 nM when using 50 μl plasma. The accuracy of the assay from plasma samples spiked with 1μM SA was 91% and with 10 μM SA was 84%. Precision was measured in five replicate samples of plasma and livers from animals administered SA. For plasma, the coefficient of variation was 6%. We also performed precision measurements in homogenized liver samples. Liver was chosen as a representative tissue for validation of tissue measurements, because in our previous experience with quantitation other small molecules, this tissue matrix tends to have the most interfering compounds that hamper sensitivity and reproducibility. When we performed the assay on homogenates of five small (<10 mg) pieces each of two different livers (one with a lower concentration SA (sample A) and one with a higher concentration SA (sample B)), we found that the coefficient of variation was 31% (19 ± 6 mol/kg tissue) and 49% (68 ± 33 mol/kg tissue), respectively. To determine if this high variation was due to heterogeneity in SA distribution in the liver or due to poor precision of the assay for tissue samples, we performed the same work-up on five replicate aliquots of a homogenate from a large (~50 mg) piece of each of the same livers. In this case, we found the coefficient of variation to be 9% (20 ± 2 mol/kg) and 7% (85 ± 6 mol/kg), respectively. Thus, heterogeneity of SA distribution in liver samples accounts for the wide variation in small pieces. We therefore used only relatively large section of tissue for subsequent analysis. 

### Pharmacokinetic parameters of SA in mice.

**Figure 5:**
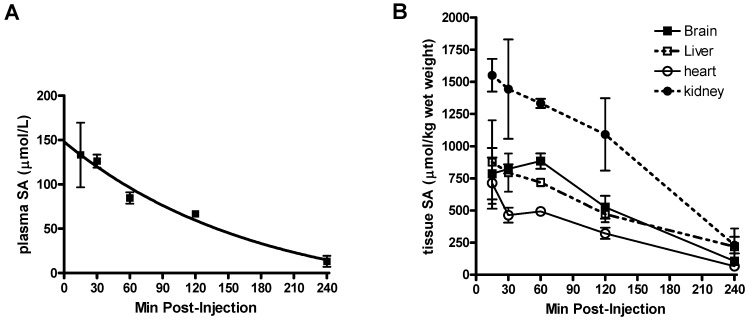
Time-dependence of SA concentration in plasma and tissue. SA was injected 200 mg/kg intraperitioneally and remaining concentration of SA (Mean ± range, 2 mice per time point) determined at various time-points by LC/MS/MS. **A)** Concentration-time curve of SA in plasma. **B)** Concentration-time curve of SA in tissue.

To determine the pharmacokinetics of SA in mice, we injected 200 mg/kg SA intraperitoneally in C57BL6 mice and measured plasma and tissue concentration at 15, 30, 60, 120, and 240 minutes post-injection (Figure 5A). SA was rapidly absorbed into the systemic circulation with maximal plasma concentrations achieved within 15 min. Plasma concentrations of SA declined exponentially yielding a half-life (t_1/2_) of 62 min. The apparent volume of distribution (V/F_app_), where the bioavailability after intraperitoneal administration is assumed to be complete (F_app_), was estimated to be 0.16 L, while the apparent plasma clearance (CL/F_app_) was 1.6 ml/min. Consistent with the large volume of distribution, tissue concentration of SA were higher than in plasma, with highest concentrations in the kidney (Figure 5B). 

The concentration of SA in plasma and tissue after IP injection spanned the range of SA that is protective in cell culture experiments. For instance, 100 μM SA inhibits formation of γKA adducts by >60% in platelets [15]. SA concentration of 10-100 μM protected against sodium channel dysfunction induced by oxidants in HEK293 cells transfected with human cardiac sodium channel (Na_V_1.5) or in the atrial-tumor derived myocyte cell line HL-1 [17]. 500 μM SA is sufficient to protect HepG2 cells against oxidant induced cell death [15].

### Feasibility of salicylamine administration in drinking water

**Figure 6:**
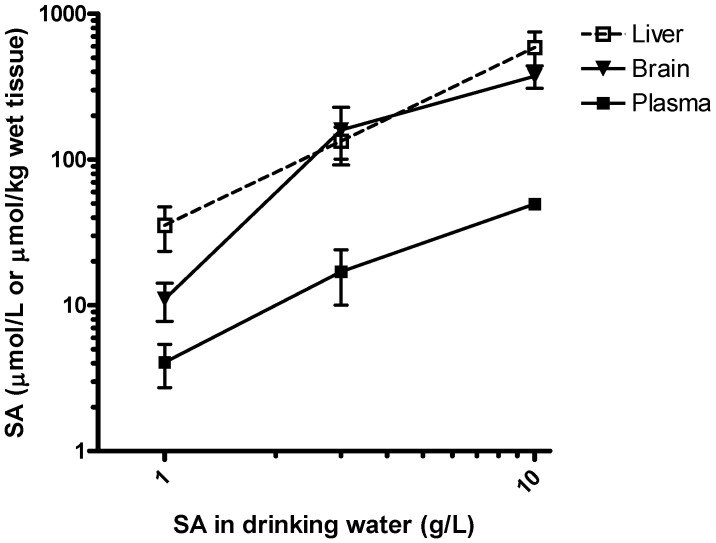
Tissue concentration of SA as a function of SA drinking water concentration.Mice were given SA at 1, 3, and 10 g/L in drinking water for 7 days and the level of SA (Mean ± SEM) in plasma, brain, and liver measured by LC/MS/MS.

γKA protein adducts are increased in a wide range of chronic diseases that may require long-term scavenger treatment to prevent protein adduct accumulation. Repeated administration of SA by intraperitoneal injection would be less desirable for long-term studies than oral delivery in food or drinking water. As the acetate salt of SA is readily soluble in water, we determined the feasibility of administering SA in drinking water. We fed mice 1, 3, and 10 g SA / L drinking water for 7 days and then measured SA concentrations in plasma and tissue. Increasing concentrations of SA in the drinking water dose-dependently increased SA concentration in plasma and tissues (Figure 6). Mice given 1 and 3 g/L SA consumed similar volumes of water per day as animals given unmedicated water. At the highest dose administered, 10 g/L, mice showed signs of toxicity including decreased volume of water consumed per day, loss of hair and body weight, and also hunching behavior. We estimated that the mice drank approximately 4.5 ml of water per 24 hour period for the 1 g/L dose (25 µmoles of SA), so that the bioavailability of SA administered in drinking water is ~38% of that delivered intraperitoneally. The lower bioavailability of orally delivered SA suggests significant first pass clearance of SA. While the concentrations of SA obtained by water supplementation at 1 and 3 g/L were somewhat lower than what was obtained by IP injection, they were still within the range expected to be therapeutic.

## 4. Conclusions

The LC/MS/MS assay developed for this study is a sensitive, specific, and accurate method to measure SA in biological samples. This assay could be used to routinely monitored SA levels even in the small amount of plasma available from live rodents during long-term treatment studies. Using this assay, we demonstrated that the plasma and tissue levels of SA obtained after intraperitoneal injection or supplementation of drinking water are in the appropriate range established by cultured cell experiments [15,17]. We also demonstrated rapid distribution of SA to plasma and all organs including brain, confirming that SA can cross the blood brain barrier. Therefore either route of SA administration could be used, based on the needs of the individual study, to elucidate the role of γKAs in conditions associated with inflammation and oxidative stress. Administration of SA in drinking water may be less onerous than repeated intraperitoneal injections for examining the role of γKA in animal models of chronic diseases like Alzheimer’s Disease, where continuous elevation in lipid peroxidation and γKA formation over long time periods is likely, so that administration of SA over similarly long time periods may be required for efficacy. 
